# The *Enterococcus faecium* Enterococcal Biofilm Regulator, EbrB, Regulates the *esp* Operon and Is Implicated in Biofilm Formation and Intestinal Colonization

**DOI:** 10.1371/journal.pone.0065224

**Published:** 2013-05-31

**Authors:** Janetta Top, Fernanda L. Paganelli, Xinglin Zhang, Willem van Schaik, Helen L. Leavis, Miranda van Luit-Asbroek, Tom van der Poll, Masja Leendertse, Marc J. M. Bonten, Rob J. L. Willems

**Affiliations:** 1 Department of Medical Microbiology, University Medical Center Utrecht, Utrecht, The Netherlands; 2 Center for Infection and Immunity Amsterdam, Academic Medical Center, Amsterdam, The Netherlands; University Hospital Schleswig-Holstein, Germany

## Abstract

Nowadays, *Enterococcus faecium* is one of the leading nosocomial pathogens worldwide. Strains causing clinical infections or hospital outbreaks are enriched in the enterococcal surface protein (Esp) encoding ICE*Efm1* mobile genetic element. Previous studies showed that Esp is involved in biofilm formation, endocarditis and urinary tract infections. In this study, we characterized the role of the putative AraC type of regulator (locus tag EfmE1162_2351), which we renamed *ebrB* and which is, based on the currently available whole genome sequences, always located upstream of the *esp* gene, and studied its role in Esp surface exposure during growth. A markerless deletion mutant of *ebrB* resulted in reduced *esp* expression and complete abolishment of Esp surface exposure, while Esp cell-surface exposure was restored when this mutant was complemented with an intact copy of *ebrB*. This demonstrates a role for EbrB in *esp* expression. However, during growth, *ebrB* expression levels did not change over time, while an increase in *esp* expression at both RNA and protein level was observed during mid-log and late-log phase. These results indicate the existence of a secondary regulation system for *esp*, which might be an unknown quorum sensing system as the enhanced *esp* expression seems to be cell density dependent. Furthermore, we determined that *esp* is part of an operon of at least 3 genes putatively involved in biofilm formation. A semi-static biofilm model revealed reduced biofilm formation for the EbrB deficient mutant, while dynamics of biofilm formation using a flow cell system revealed delayed biofilm formation in the *ebrB* mutant. In a mouse intestinal colonization model the *ebrB* mutant was less able to colonize the gut compared to wild-type strain, especially in the small intestine. These data indicate that EbrB positively regulates the *esp* operon and is implicated in biofilm formation and intestinal colonization.

## Introduction

For long, the Gram-positive species *Enterococcus faecium* was considered a harmless commensal of the mammalian intestinal tract. However, in the last two decades *E. faecium* emerged as one of the leading nosocomial pathogens [1]. Molecular epidemiological studies using multilocus sequence typing (MLST) identified host-specific genogroups including three hospital-associated *E. faecium* (HA-*Efm*) lineages designated lineage-17, -18 and -78 [1]–[4]. HA-*Efm* are characterized by multidrug-resistance including ampicillin and quinolone resistance and enrichment of several (putative) virulence genes [5]–[8], including a gene encoding the approximately 200 kDa surface protein Esp, which is located on a pathogenicity island (PAI) [9]. Recently, we showed that this PAI is self-transmissible and contained the characteristics of an integrative conjugative element [10], and was therefore renamed in ICE*Efm1*. By constructing an *esp* insertion-deletion mutant (E1162Δ*esp:cat*) and by using different animal models, we and others demonstrated that Esp is involved in biofilm formation [12] and contributes to the pathogenesis of urinary tract infection [13] and endocarditis [14]. Furthermore, van Wamel *et al.* [15] demonstrated variable cell surface expression of Esp among different strains, growth condition (temperature and presence of air oxygen) dependent expression of Esp and a correlation between expression levels and initial adherence and biofilm formation in a polystyrene binding assay. Further analysis of ICE*Efm1* encoded genes identified upstream of *esp* an ORF with locus tag EfmE1162_2351 with similarity to the family of AraC type of transcriptional regulators. Here we report functional analysis of EfmE1162_2351, which we renamed *ebrB* for enterococcal biofilm regulator B and performed additional analysis to determine dynamics of Esp expression.

## Materials and Methods

### Ethics Statement

The Animal Care and Use Committee of the University of Amsterdam approved the mouse intestinal colonization experiment.

### Bacterial strains, plasmids and growth conditions

*E. faecium* and *Escherichia coli* strains and plasmids used in this study are listed in Table 1. The *E. coli* strains DH5α (Invitrogen) and EC1000 [16] were grown in Luria-Bertani (LB) medium. Enterococci were grown in brain heart infusion (BHI) medium, Tryptic soy broth supplemented with 1% glucose (TSBg) or Tryptic soy agar supplemented with 5% sheep red blood cells (BA) (BD, Alphen aan den Rijn, The Netherlands) at 37°C. For enterococci, the antibiotics gentamicin and spectinomycin were used in concentrations of 300 µg/ml. For *E. coli*, gentamicin and spectinomycin were used in concentrations of 30 µg/ml and 100 µg/ml, respectively. All antibiotics were obtained from Sigma-Aldrich (Saint Louis, MO). The genome sequence of *E. faecium* strain E1162 is available (GenBank: ABQJ00000000), including the *esp* containing ICE*Efm1* encoded on contig156 (ABQJ01000139).

**Table 1:** Bacterial strains and plasmids.

Strain or plasmid	Relevant characteristics a	Reference or source
Strains		
*E. faecium*		
E1162	Clinical blood isolate; AmpR, VanS, ChlS, GenS, SpcS; ICE*Efm1*+	[11]
E1162Δ*ebrB*	Markerless deletion mutant of *ebrB* of E1162; GenS; ICE*Efm1+*	This study
E1162Δ*ebrB+*pEF25	Complementation strain of E1162Δ*ebrB,* harboring "empty" vector pEF25; SpcR, GenS; ICE*Efm1*+	This study
E1162Δ*ebrB+ebrB*	Complementation strain of E1162Δ*ebrB*, harboring pEF25-*ebrB*; SpcR, GenS; ICE*Efm1+*	This study
E1162Δ*esp*	Markerless deletion mutant of *esp* of E1162; GenS; ICE*Efm1+*	This study
E1162Δ*esp+*pEF25	Complementation strain of E1162Δ*esp,* harboring "empty" vector pEF25; SpcR, GenS; ICE*Efm1*+	This study
E1162Δ*esp+esp*	Complementation strain of E1162Δ*esp*, harboring pEF25-*esp*; SpcR, GenS; ICE*Efm1+*	This study
E0305	Hospital outbreak isolate, *ebrB* natural mutant by insertion of IS*256*	[24]
E0305+pEF25	Complementation strain of E0305, harboring "empty" vector pEF25	This study
E0305+*ebrB*	Complementation strain of E0305, harboring pEF25-*ebrB*	This study
*E. coli*		
DH5α	*E. coli* host strain for routine cloning	Invitrogen
EC1000	MC1000 *glgB*:*repA*	[16]
Plasmids		
pWS3	Shuttle plasmid; ts in gram-positive hosts; SpcR	[21]
pEF39	pWS3: *ebrB* fusion with gentamicin resistance casssette cloned in the EcoRI site of the *ebrB*gene fusion fragment	This study
	plasmid for generating an *ebrB* marked mutation; SpcR, GenR	
pEF40	pWS3: *esp* fusion with gentamicin resistance casssette cloned in the EcoRI site of the *esp*gene fusion fragment	This study
	plasmid for generating an *esp* marked mutation; SpcR, GenR	
pWS3-Cre	pWS3 derivative expressing Cre in *E. faecium*	[20]
pAT18	shuttle plasmid; EryR	[22]
pEF25	shuttle plasmid pAT18 with spectinomycin resistance cassette cloned in the EcoRI site; SpcR, EryR	This study
pEF25-*esp*	Complementation plasmid for *esp;* pEF25 carrying gene *esp*; SpcR, EryR	This study
pEF25-*ebrB*	Complementation plasmid for *ebrB;* pEF25 carrying gene *ebrB*; SpcR, EryR	This study

a Amp, ampicillin; Van, vancomycin; Chl, chloramphenicol; Gen, gentamycin; Spc, spectinomycin; ICE*Efm1+*, *E. faecium ebrB* containing pathogenicity island

### Standard molecular techniques

Plasmid DNA purification (Qiagen, Venlo, The Netherlands), digestion with restriction endonuclease (New England Biolabs, Leusden, The Netherlands), amplification of DNA by PCR performed in 25 µl volumes with HotStarTaq Master Mix (Qiagen, Venlo, The Netherlands), AccuPrime Taq DNA polymerase High Fidelity with buffer 1 (Invitrogen, Breda, The Netherlands) or Expand Long Template PCR system with buffer 3 (Roche Applied Sciences, Almere, The Netherlands) and ligation of DNA fragments with T4 DNA ligase (Invitrogen) were performed according to the manufacturers' instructions. *E. faecium* chromosomal DNA was purified using the Wizard Genomic DNA purification kit (Promega, Leiden, The Netherlands) according to the protocol with minor modifications. In brief, 1 ml of overnight culture was harvested by centrifugation and resuspended in 580 µl 50mM EDTA. Lysozyme (20 µl, 50 mg/ml) was added and the suspension was incubated at 37°C for 1 h. The sequential steps were according to the manufacturers' protocol. Primers were purchased from Invitrogen and are listed in Table 2. PCRs were performed with a 9800 Fast Thermal Cycler (Applied Biosystems, Life technologies, The Netherlands). PCR amplification conditions using HotStarTaq and performed in a volume of 25 µl were as follows: initial denaturation at 95°C for 15 min, followed by 30 cycles of 30 s at 94°C, 30 s at 53°C and 72°C (the time depending on the size of the PCR product). For AccuPrime™ Taq polymerase the following PCR conditions were used, initial denaturation at 94°C for 2 min, followed by 30 cycles of 30 s at 94°C, 30 s at 53°C and 68°C for 1 min per kb of PCR product. The PCR conditions for the Expand Long Template PCR systems included an initial denaturation at 95°C for 2 min, followed by 10 cycles of 10 s at 94°C, 30 s at 53°C and 68°C (the time depending on the size of the PCR product), followed by 25 cycles with the same denaturing and annealing and elongation conditions, with an increased elongation time of 20 s per cycle. Plasmids were introduced into *E. faecium* by electroporation using a Gene Pulser unit (Bio-Rad Laboratories, Richmond, CA) as described elsewhere [17].

**Table 2:** Primers used in this study.

Primer name	Primer sequencea,b	Startposition^c^
pAW068-spcF	5′-GGAATTCTTTTGTTTCGAAGCAGCAGAT	
pAW068-spcR	5′-GGAATTCGGACGCTTTATTCTTCCCAAA	
ebrB-1F	5′-TCTGTCGTTCAATTCATCG	69605
ebrB-2F	5′-CGGATCATAATAATTATTGTCTTTG	68488
ebrB-1R	5′-GTCATATTCATTTAACACACTATTATTACC	70088
esp-1R	5′-AATACTCTCTTATTATTCTTGCTAACC	71021
hyp-1R	5′-ATTGGAGTTATCAACATTTTTTC	70515
Deletion/complementation		
ebrBUp-F-XhoI	5′-CCGCTCGAGCATATATCTTCTTAAATATCAAACATG	68219
ebrBUp-R-EcoRI	5′-AGTATGGTTTGAATTCTAATAAGACTTCTTTATCTGAAAACAC	68747
ebrBDn-F-EcoRI	5′-CTTATTAGAATTCAAACCATACTATCAGTGAAGTTTC	69784
ebrBDn-R-SmaI	5′-CCCCCCGGGCGTAATAATCTTCCCAGCTTTC	70302
ebrB-check up	5′-GTATTAGCGGTGTTCAAAATG	67950
ebrB-check down	5′-ATTGGAGTTATCAACATTTTTTC	70515
ebrBcompF	5′-GCGGAGCTCGTTAGCTTATTTTGACAGAGGAATAG	68634
ebrBcompR	5′-GGTACGCCCGGGTCAGCTAATGTTGTTGAAATTG	69925
espUp-F-XhoI	5′-CCGCTCGAGGTTGATAACTCCAATCATTCG	70501
espUp-R-EcoRI	5′-GATTGTCAGGAATTCTCTCTTATTATTCTTGCTAACCAT	71017
espDn-F-EcoRI	5′-ATAATAAGAGAGAATTCCTGACAATCAAGGTAGCAAC	76742
espDn-R-SmaI	5′-CCCCCCGGGCTCAGAATTTAGTGTCATTCTATTTG	77264
esp-check up	5′-TCTGTCGTTCAATTCATCG	69605
esp-check down	5′-ATGTATTCCATTTTTTGATAGTATTTC	77674
espcompF	5′-CCGGAATTCGCTTGCATCAAAATAAACTACATGGGTATAAT	70993
	AGCAATGAAATGCATTTCAAAAATATTTTGAGGAGAATTT	
	AGTATGGTTAGCAAGAATAATAAGAG	
espcompR	5′-CCGGAATTCCCTCTTTTCAGAGAAGATT	76954
qRT-PCR		
ebrB-RT-F	5′-TGAGGGATTCTGGGATTGTTT	69528
ebrB-RT-R	5′-GCCGATGAATTGAACGACAGA	69625
esp-RT-F	5′-CCACGAGTTAGCGGGAACAG	72498
esp-RT-R	5′-TTGGAGCCCCATCTTTTTCA	72599
nox-RT-F	5′-AGCCGCAGCTCGATTTCTAA	77095
nox-RT-R	5′-AACGATGTCCCACATTCCAA	77193
mur-RT-F	5′-GGTGAGCCGATTCATGCAGT	79670
mur-RT-R	5′-AACGCGGTTGATCCATCTTC	79776
1542-RT-F	5′-TGGTCACCTTACTGTTGTTGAGGA	80323
1542-RT-R	5′-CGTTTCATTCCCACAGTCACA	80404
efflux-RT-F	5′-ACGGGTGGTACAAGCCATTG	81857
efflux-RT-R	5′-GCCCGACCACGTTCATGTAT	81949
tufA-RT-F	5′-TACACGCCACTACGCTCAC	
tufA-RT-R	5′-AGCTCCGTCCATTTGAGCAG	
5′-RACE		
ebrB-GSP1	5′-CAGACCGAATCGTATCTCCA	69383
ebrB-GSP2	5′-ATCAGCCATTGCAACATTCA	69043
esp-GSP1	5′-GGTTTGCGTATCGGTTGTTT	71577
esp-GSP2	5′-TTCTGCCCCAGCAAATAATC	71559

a Restriction sites are boldface

b Regions -35, -10, the ribosome binding site from the *bacA* promoter and the ATG startcodon of *esp* are underlined [12]

c Nucleotide reference positions relative to ABQJ01000139

### Bioinformatic analysis

The putative helix-turn-helix (HTH) motif of EbrB encompassing amino acids 345-386 was aligned with a sequence logo generated using a Prosite database containing 310 HTH motifs (accession number PS00041) in Weblogo, Version 2.8.2 [18]. Presence of putative transcription terminators were predicted using RNAfold (http://rna.tbi.univie.ac.at/) [19].

### Generation of targeted markerless deletion mutants

Recently, in our group the first targeted markerless deletion mutants were constructed based on the Cre-*lox* system [20], [21]. Here, we used this method to generate markerless deletion mutants of the *esp* and *ebrB* gene. Previously, Heikens *et al.* constructed an *esp* insertion-deletion mutant E1162Δ*esp:cat* [12]. In order to compare Esp expression levels with the newly generated E1162Δ*ebrB* independently of a putative effect of the inserted chloramphenicol cassette, we also constructed a markerless deletion mutant in *esp*. For the amplification of the 5′-flanking regions of *ebrB* and *esp*, we used primers ebrBUp-F-XhoI, ebrBUp-R-EcoRI, espUp-F-XhoI and espUp-R-EcoRI, respectively, while for the 3′-flanking regions primers ebrBDn-F-EcoRI, ebrBDn-R-SmaI, espDn-F-EcoRI and espDn-R-SmaI were used (Table 2). Generation of a marked deletion mutant was performed as described [20] and was confirmed by PCR using the *ebrB* and *esp* check up and check down primers (Table 2). Removal of the gentamicin resistance marker was obtained by electroporation of pWS3-Cre into the marked deletion mutants as described [20]. Loss of the marker was confirmed by PCR using the *ebrB* and *esp* check up and check down primers.

### *In trans* complementation of mutants

A modified pAT18 vector [22] designated pEF25 was used for complementation studies (Table 1). In this vector a spectinomycin resistance cassette amplified from vector pAW068 [23] using primers pAW068-spcF and pAW068-spcR both containing EcoRI restriction sites, was cloned into the EcoRI site of pAT18.

To complement E1162▵*ebrB*, *ebrB* was amplified from E1162 genomic DNA using AccuPrime™ Taq Polymerase with primers ebrBcompF and ebrBcompR (Table 2). The forward primer, which contained a SacI restriction site to facilitate cloning of the fragment, was located 86 bp upstream the startcodon of *ebrB*, to ensure that expression of *ebrB* is driven by its native promoter, which was mapped by 5′ RACE as described below. The reverse primer included a SmaI restriction site. The resulting *ebrB* containing PCR product was digested with SacI and SmaI and ligated to a similar digested pEF25 resulting in pEF25-*ebrB*. The recombinant plasmid pEF25-*ebrB* and the negative control pEF25 were introduced into the E1162▵*ebrB* by electroporation resulting in E1162?*ebrB*+*ebrB* and E1162?*ebrB*+pEF25, respectively (Table 1).

A screen for the presence of *ebrB* using primers ebrB-2F and ebrB-1R (Table 2) on a selection of isolates revealed that strain E0305 [24] was a natural mutant for *ebrB* as it contained an insertion of IS*256* in *ebrB* as determined by sequencing of the obtained PCR fragment using the same primers ebrB-2F and ebrB-1R. To complement E0305 with an intact copy of *ebrB*, we also introduced pEF25 and pEF25-*ebrB* by electroporation resulting in E305+pEF25 and E0305+*ebrB*, respectively (Table 1).

Complementation of the E1162▵*esp* was performed as described for the *ebrB* mutant using primers espcompF, including the promoter regions of the *bacA* gene of *Enterococcus faecalis* and espcompR as described previously [12]. Several attempts to transform the ligation mixture in *E. coli* failed, which was also reported for other large repeat containing genes [25] though direct electroporation of the ligation mixture in the E1162▵*esp* resulted in stable complementation. The complemented strain of the E1162▵*esp* mutant was designated E1162▵*esp*+*esp* (Table 1). For comparison E1162▵*esp* was complemented with the empty vector pEF25 resulting in E1162▵*esp*+pEF25 (Table 1).

### Determination of growth curves

A BioScreen C instrument (Oy Growth Curves AB, Helsinki, Finland) was used to monitor effects of *esp* or *ebrB* deletion on bacterial growth. Wild-type *E. faecium* E1162 and mutants were grown overnight in BHI and TSBg, while the complemented strains were grown in BHI and TSBg with the addition of spectinomycin. Cells were inoculated at an initial OD_660_ of 0.0025 into 300 µl BHI and TSBg and incubated in the Bioscreen C system at 37°C with continuous shaking and absorbance of 600 nm (A_600_) was recorded every 15 min for 9 hours. Each experiment was performed in triplicate.

### Determination of (cell-surface) expression of Esp

Dynamics of Esp (cell surface) expression was determined for wild-type E1162, E1162▵*ebrB,* E1162▵*esp*, E0305 and the complemented strains grown on blood agar plate and in broth, including BHI and TSBg using flow cytometry, electron microscopy and Western blotting. Flow cytometry, electron microscopy and Western blotting were performed as previously described [12], [15]. Flow cytometry experiments were performed in triplicate.

### Transcriptome profiling

In order to compare the transcriptomes of *E. faecium* E1162 and E1162▵*ebrB*, microarray analysis was performed on four independent biological replicates using a custom made 8×15K Agilent *E. faecium* E1162 microarray as previously described [20]. E1162 and E1162▵*ebrB* were grown in TSBg for 18 hours. Cultures were then diluted to OD_660_ 0.025 in 20 ml of prewarmed TSBg and grown to OD_660_ 0.3. RNA isolation, cDNA synthesis, labeling and hybridization were performed as previously described [20]. After removal of the data for the different controls printed on the microarray slides, the normalized data for each spot from the microarrays were analyzed for statistical significance using the Web-based VAMPIRE microarray suite [26], [27]. A spot was found to be differentially expressed between two samples using the threshold of a false discovery rate smaller than 0.05. Changes of ≥ 2-fold for up- and down regulated genes in the parental strain were introduced as additional significance limits. A gene with two identical probes or all four probes meeting this criterion were classified as differentially expressed. The microarray data generated in this study have been deposited in the ArrayExpress database (http://www.ebi.ac.uk/arrayexpress) under accession number E-MEXP-3801.

### Reverse transcription and (quantitative) real-time RT-PCR

**Figure 1:**
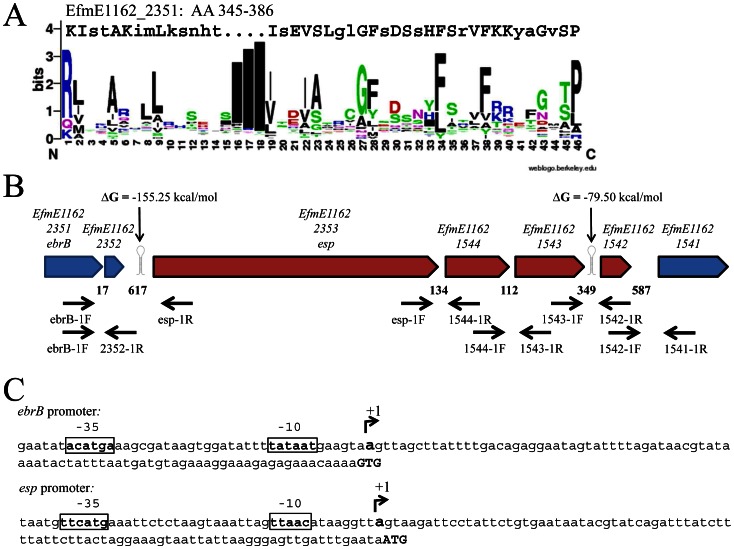
Structural organization of the *ebrB* region. (A) Alignment of the putative helix-turn-helix motif, amino acids 345-386, of EbrB to a sequence logo generated using a Prosite database containing 310 HTH motifs (accession number PS00041) in Weblogo. Conserved amino acids are depicted in capital letter. (B) Overview *ebrB* region, including the *esp* operon in red, primer sites and predicted transcription terminators (RNAfold). Numbers indicate the intergenic distances in bp (C) *ebrB* and *esp* promoter mapping, in bold/box putative -35 and -10 sequence and transcription start (+1).

In all cases, RNA was isolated as described for the microarray. In order to investigate whether *esp* and four downstream of *esp* located genes with locus-tags EfmE1162_1544, EfmE1162_1543, EfmE1162_1542 and EfmE1162_1541 are part of an operon, we isolated RNA from wild-type strain E1162 (four biological replicates), performed first strand synthesis using Maxima reverse transcriptase (Fermentas, Thermo Scientific, St. Leon-Rot, Germany) in combination with 5′-end located gene specific primers 1544-1R, 1543-1R, 1542-1R and 1541-1R, respectively (Table 2, Fig. 1B) on each individual gene. The presence of intergenic cDNA was subsequently determined by PCR using the same gene specific primer in combination with a 3′-end located primer of its upstream located gene, i.e. esp-1F/1544-1R, 1544-1F/1543-1R, 1543-1F/1542-1R and 1542-1F/1541-1R (Fig.1B). As negative control the same procedure for cDNA synthesis was followed but without adding reverse transcriptase. As positive control for the PCR we included purified genomic E1162 DNA.

Quantitative real time PCR (qRT-PCR) was used to confirm the microarray data, growth condition dependent expression of *esp* and *ebrB* in wild-type strain E1162 and to determine whether *esp* is part of an operon by comparison of differential expression of *esp* and its previously mentioned four downstream of *esp* located genes, between strains E1162▵*ebrB*+*ebrB* and E1162▵*ebrB*+pEF25 and E1162▵*esp*+*esp* and E1162▵*esp*+pEF25. cDNA was synthesized from RNA using Maxima First strand cDNA synthesis kit for RT-qPCR (Fermentas, Thermo Scientific, St. Leon-Rot, Germany) and 1 µg of total RNA. Quantitative PCRs using primers indicated with RT (Table 2) on the synthesized cDNAs were performed using Maxima® SYBR Green/ROX qPCR Master Mix (Fermentas) using a StepOne™ Realtime PCR system (Applied Biosystems, Nieuwekerk a/d IJssel, The Netherlands) with the following program: 95°C for 10 min, and subsequently 40 cycles of 95°C for 15 sec, 55°C for 1 min. The expression of the *tufA* gene was used as a reference for the determination of relative expression levels (Table 2) [28]. For the confirmation of the microarray data and the analysis of the *esp* operon, relative transcript levels were calculated by using the relative expression software tool (REST (Qiagen)) [29]. For the growth condition dependent expression of *esp* and *ebrB*, we normalized the Ct values of each sample by the amplification efficiency. The relative transcript level (fold difference relative to *tufA*) of the *esp* and *ebrB* genes were calculated by using the normalized Ct value of the *tufA* housekeeping control minus the normalized Ct value of the *esp* and *ebrB* genes. The resulting value represents a log2 transformed fold difference in gene expression. Statistical significance between wild-type and mutant was assessed by the unpaired two-tailed Student′s *t*-test. This experiment was performed with two biological replicates and qRT-PCR performed in duplo.

### Promoter mapping of *ebrB* and *esp* using 5′RACE

Total RNA was isolated as previously described [20]. We used the 5′ RACE kit (Rapid amplification of cDNA ends, Invitrogen, The Netherlands) to map the promoter of *ebrB* and *esp* according to the manufacturers′ protocol. After first strand synthesis using gene specific primers 1 (GSP1) (Table 2), a nested PCR with GSP2 primers was performed to amplify the product and cloned in pGEM-T Easy TA cloning vector (Promega, Madison, WI). Inserts were sequenced to determine the cDNA end.

### Biofilm semi-static model and confocal laser scanning microscopy (CLSM)

For the semi-static biofilm model, overnight in TSBg grown wild-type E1162, E1162Δ*esp* and E1162Δ*ebrB* and complemented strains E1162▵*esp*+*esp,* E1162▵*esp*+pEF25, E1162▵*ebrB*+*ebrB* and E1162▵*ebrB*+pEF25 were diluted to OD_660_ 0.01 in a 6-well polystyrene plate (Corning Inc.) containing a poly-L-lysin coated coverslip (0.45 µm; diameter, 12 mm; Becton Dickinson) and 6 ml TSBg to facilitate attachment and biofilm formation. The 6-well plates were incubated at 37°C under gently shaking at 120 rpm, for 24 h or 72 h. The coverslips were washed two times with 0.85% NaCl, and the biofilms were chemically fixed using 8% glutaraldehyde (Merck) in 0.85% NaCl for 20 min and washed again two times with 0.85% NaCl. The biofilms were stained using 15 µg/ml propidium iodide (PI) in 0.85% NaCl for 15 min. After incubation, the stain was removed and coverslips were transferred to glass microscope slides. Biofilms were analyzed by a confocal laser scanning microscope (CLSM) (Leica SP5) equipped with an oil plan-neofluor 63x/1.4 objective. PI was excited at 633 nm. Z-stacks were taken with an interval of 0.42 µm. Pictures were analyzed with LAS AF software (Leica) and biofilm formation was quantified using Comstat (Heydorn et al., 2000)/Matlab R2010b software (The MathWorks). The average thickness and total biomass of the biofilms was measured at five randomly chosen positions.

### Biofilm flow cell model

Dynamics of biofilm formation were studied in a Stovall flow cell system (Life Science, Inc., Greensboro, N.C.) for E1162 (wild-type), E1162Δ*esp* and E1162Δ*ebrB* in TSB diluted in PBS (1:10, v:v) with 1% glucose. After inoculation of the flow chambers bacterial cells were allowed to adhere for 1h in the absence of flow. Biofilms were grown under a flow of 0.13 ml/min for 17 h. Biofilm development was scanned at regular intervals of 7 min (40×objective), with a DFC360 FX Digital Camera Kit SP5 (Leica) using CLSM (Leica SP5).

### *In vivo* colonization model

Intestinal colonization by wild-type E1162 and E1162Δ*ebrB* was tested as previously described [30], but with a modification of the decolonization regimen as recently described by Zhang *et al.* [31]. In brief, specific pathogen free 10-week-old male Balb/c mice (16 mice in total) were purchased from Charles River Laboratories Inc. (Maastricht, the Netherlands) and housed as described previously. Two days before inoculation of bacteria, mice were administered subcutaneous injections of ceftriaxone (Roche, Woerden, The Netherlands; 100 µl per injection, 12 mg/ml) two times daily and one time at the day of inoculation. For the remaining duration of the experiment, cefoxitin (0.125 g/l) was added to sterile drinking water. The inoculum of 2×10^9^ CFU/300 µl Todd Hewitt Broth E1162 or E1162Δ*ebrB* was prepared as described previously [30]. Collection of samples and determination of bacterial outgrowth was performed as previously described [30].

### Statistical analysis

For analysis of cell surface expression of Esp, biofilm formation and intestinal colonization an unpaired two-tailed Student's *t*-test was applied.

## Results

### Bioinformatic analysis of EbrB

Upstream of *esp* we identified a gene, *efmE1162_2351*, renamed *ebrB* for reasons described later, which was annotated to belong to the AraC family of transcriptional regulators. BLAST analysis identified an AraC type helix-turn-helix (HTH) motif in the C-terminal region of the protein. These HTH domains are known to be involved in binding to promoter regions of genes [32], [33]. Comparison of the putative HTH motif encompassing amino acids 345-386 with a sequence logo generated using a Prosite database containing 310 HTH motifs identified 24 conserved amino acids known to be important for the structure of the helices (Fig. 1A) [34]. Furthermore, EbrB was also annotated to belong to the TIGR04094 protein family of transcriptional regulators that are located adjacent to proteins with the YSIRK variant form of signal peptide. Indeed, the downstream located Esp encoding gene has a variant YSIRK motif, YSIKK, in its signal peptide. Based on these genetic features, we hypothesized that EbrB is involved in the transcriptional regulation of *esp* expression.

### Transcriptional organization of the *ebrB-esp* region

To investigate the transcriptional organization of the *ebrB*-*esp* region, we first determined whether *ebrB* and *esp* are transcribed as a single RNA molecule. A PCR using primers ebrB-1F and esp-1R (Fig. 1B, Table 2) on synthesized cDNA obtained from E1162 RNA yielded no product (data not shown). A positive PCR result was obtained on cDNA using primers in *ebrB* (ebrB-1F ) and in a downstream located small coding sequence (locus tag EfmE1162_2352) of unknown function (2352-1R) (Fig. 1B, Table 2, data not shown). These PCR results in combination with the presence of a predicted transcription terminator downstream the hypothetical CDS with a ▵G of -155.25 kcal/mol (Fig. 1B), indicated that *erbB* and *esp* are not part of the same operon. The transcription start sites of *ebrB* and *esp* were identified at 86-bp and 89-bp upstream their respective startcodon using 5′-RACE analysis (Fig. 1C). A putative promoter region, including -10 and -35 boxes were identified 99-bp and 124-bp upstream of the *ebrB* startcodon and 102-bp and 128-bp upstream of the *esp* startcodon (Fig. 1C).

### EbrB is involved in cell-surface expression of Esp

**Figure 2:**
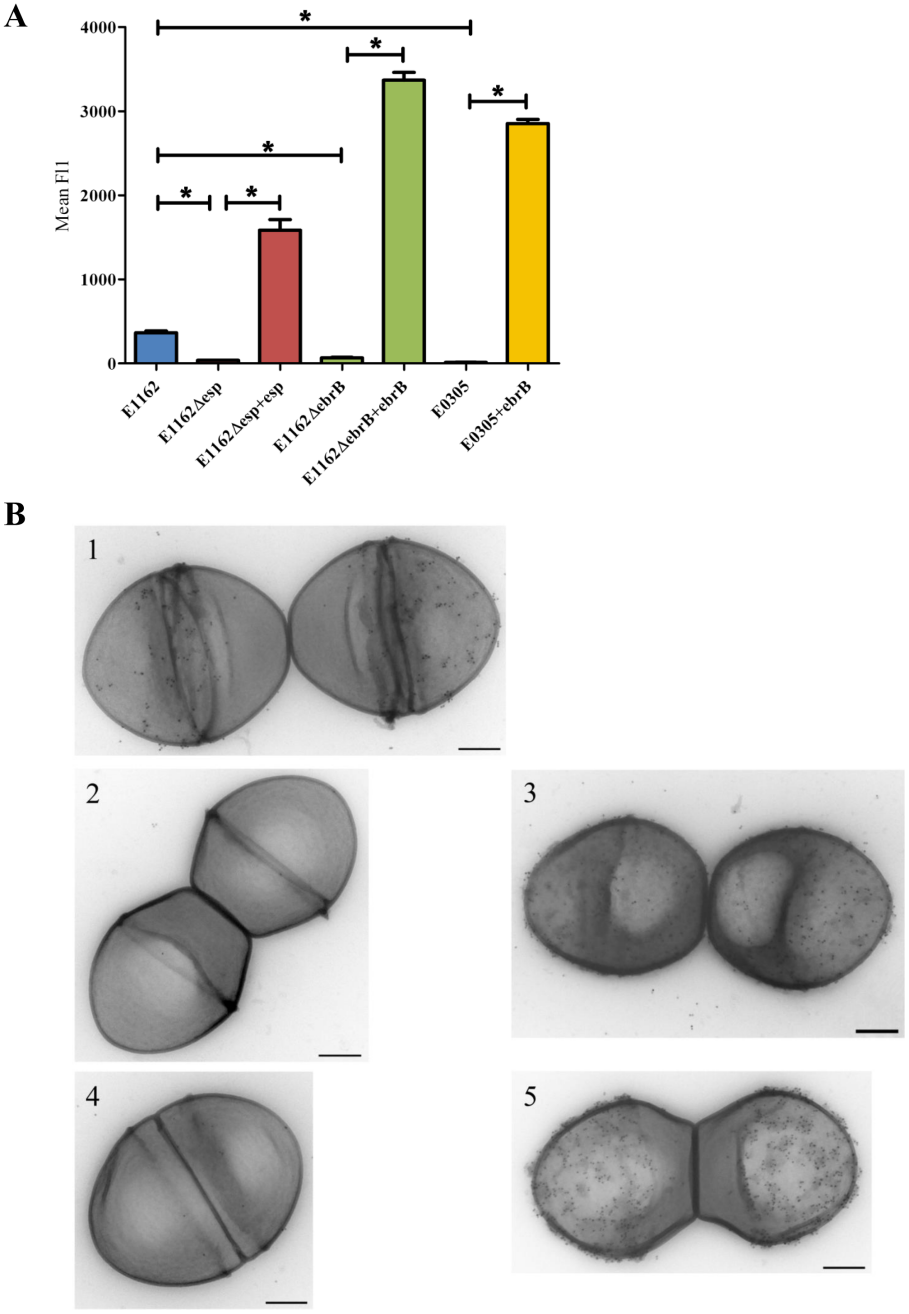
Cell surface expression of Esp. (A) Cell surface expression of Esp on plate grown cells was analyzed by flow cytometry using rabbit α-Esp immune serum for wild-type strain E1162, *esp* mutant strain (E1162▵*esp*) and the *esp* complemented strain (E1162▵*esp*+*esp*), *ebrB* mutant strain (E1162▵*ebrB*) and the *ebrB* complemented strain (E1162▵*ebrB+ebrB*) and a natural *ebrB* mutant strain (E0305) and complemented strain (E0305+*ebrB*). The means of mean Fl1 from three independent experiments are shown. Asterisks represent significant differences (*p<0.01) as determined by an unpaired two-tailed Student's *t*-test) between the indicated samples. (B) Shown are transmission electron micrographs at a magnification of 60,000x. The wild-type strain (E1162) (1), the *esp* mutant strain (E1162▵*esp*) (2), the *esp* complemented strain (E1162▵*esp* +*esp*) (3), the *ebrB* mutant strain (E1162▵*ebrB*) (4) and the *ebrB* complemented strain (E1162▵*ebrB*+*ebrB*) (5) were incubated with rabbit α-Esp immune serum, followed by protein-A-Gold. Bars, 200nm.

To investigate the role of EbrB in surface expression of Esp we constructed markerless EbrB deficient and Esp deficient mutants and determined first growth rates of these mutants. Growth curves of wild-type and the mutant strains grown in either BHI or TSBg using the Bioscreen were similar (Fig. S1). Next, wild-type E1162, E1162Δ*esp* and E1162Δ*ebrB* were grown on BA plates to determine cell-surface expression of Esp by flow cytometry using rabbit α-Esp immune serum (Fig. 2A). Esp expression was significantly reduced in both E1162Δ*esp* and E1162Δ*ebrB* compared to wild-type (*p*<0.01). Transmission electron microscopy confirmed the absence of Esp on the cell surface in both mutants (Fig. 2B). Esp expression was restored and highly expressed in the *esp* and *ebrB* complemented strains E1162Δ*esp*+*esp* and E1162▵*ebrB*+*ebrB* (Fig. 2), but not in the complemented strains with the empty vector pEF25 (data not shown), demonstrating that EbrB is involved in regulation of Esp expression.

In a previous screen for the presence of *ebrB* using *ebrB* specific primers, we identified a strain in which the *ebrB* PCR yielded a product that was 1.1 kbp larger than expected. Sequencing of this PCR product revealed that this strain, coded E0305 and originating from a hospital outbreak from Detroit area, USA [24] contained an insertion of IS*256* in *ebrB*, thus represents a natural *ebrB* mutant (data not shown). In line with observations for E1162Δ*ebrB* we also observed abolished cell surface expression of Esp in E0305, while Esp expression was restored in the *ebrB* complemented strain E0305+*ebrB* (Fig. 2A), but not in E0305+pEF25 (data not shown). All these findings support our hypothesis that EbrB regulates *esp* expression.

### Growth phase dependent cell-surface Esp expression

**Figure 3:**
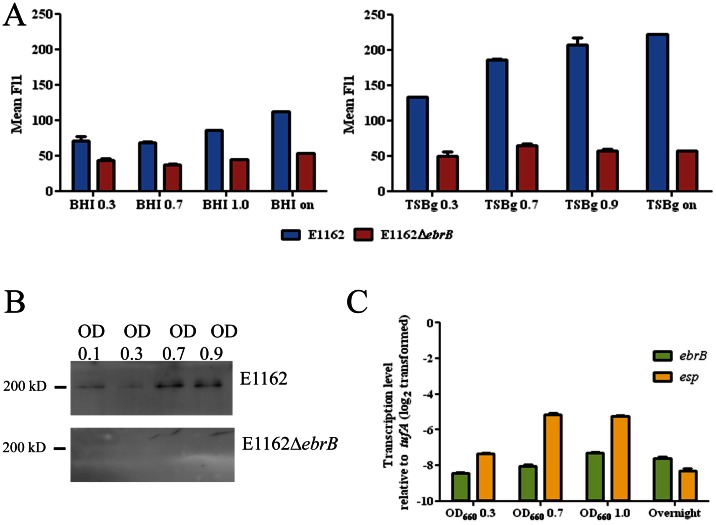
Dynamics of Esp expression. (A) Cell surface Esp expression for wild-type strain (E1162) in blue and the *ebrB* mutant strain (E1162▵*ebrB*) in red, during growth in BHI and TSBg at OD_660_ 0.3, 0.7 and 1.0 and from an overnight culture analyzed by flow cytometry using rabbit α-Esp immune serum. (B) Esp expression in E1162 and E1162▵*ebrB* cell extracts obtained during growth in TSBg at OD_660_ 0.1, 0.3, 0.6 and 0.9 analyzed by Western blot using rabbit α-Esp immune serum. (C) qRT-PCR analysis of *ebrB* and *esp* expression ratios in E1162 at OD_660_ 0.3, 0.7, 1.0 and overnight culture. The data from the qRT-PCR were normalized using *tufA* as an internal standard. The differences in gene expression (log_2_-transformed data) relative to *tufA* are shown.

Previously, van Wamel *et al*. demonstrated temperature and air-oxygen dependent cell-surface expression of Esp using BA plate grown cells [15]. In order to determine dynamics of Esp expression in different growth phases, E1162 and E1162Δ*ebrB* were grown in BHI and TSBg broth and Esp expression were measured using flow cytometry at OD_660_ 0.3, 0.7, 1.0 (BHI) or 0.9 (TSBg) and from overnight culture (Fig. 3A). Increased Esp expression was observed in both BHI and TSBg during growth, although growth in BHI resulted in only a small increase in Esp expression. In TSBg, the highest increase of Esp expression was observed between OD_660_ 0.3 and 0.7 resulting in higher levels of Esp expression in late log and stationary phase. As expected, only low background levels of fluorescence were measured in E1162Δ*ebrB.* These levels were comparable to the background levels observed in the Esp deficient mutant (data not shown), which indicates that Esp is not expressed on the cell surface of the EbrB deficient mutant. In order to determine whether the addition of glucose in TSBg explained the difference with BHI grown cells, 1% glucose was also added to BHI, but did not result in higher Esp expression levels (data not shown).

Western blot analysis using α-Esp antibodies on cell lysates from E1162 and E1162Δ*ebrB* grown in TSBg at OD_660_ 0.1, 0.3, 0.7 and 0.9 was performed to determine whether Esp might be expressed in early and mid-log phase but not transported to the cell surface. This revealed a clear increase of Esp at higher OD_660_, while Esp was absent in E1162Δ*ebrB* (Fig. 3B), which means that increased cell surface expression of Esp at higher cell densities is correlated with total Esp protein expression levels.

Dynamics of *ebrB* and *esp* expression was further investigated on RNA level using qRT-PCR on synthesized cDNA obtained from RNA extracted in the different growth phases in TSBg. Relative to *tufA*, transcription levels of both *ebrB* and *esp* were lower in all growth phases, *i.e.* for *ebrB* between 7.3- and 8.4-fold and for *esp* between 5.1- and 8.3-fold lower expression (Fig. 3C). However, while only small fluctuation in transcription levels were observed for *ebrB*, increased transcription levels of *esp* were observed at OD_660_ 0.7 and OD_660_ 1.0 compared to OD_660_ 0.3 (Fig. 3C). After overnight growth expression levels of *ebrB* and *esp* were comparable. The finding of increased *esp* transcription correlate with the increase in cell surface expression of Esp during late-log and stationary phase. Interestingly, as the higher Esp expression was found in growth phases with increased cell densities including late log and stationary phase, this finding suggests that Esp expression is cell density dependent which seems to be independent of EbrB, while *ebrB* expression did not change during increased cell density.

### Comparative analysis of the transcriptome of *E. faecium* E1162 and E1162Δ*ebrB*

In order to determine whether deletion of *ebrB* also influenced expression levels of other genes and might act as a global regulator, we used microarray-based transcriptome analysis on exponentially grown (OD_660_ = 0.3) *E. faecium* E1162 and E1162Δ*ebrB* cultures in TSBg medium. Surprisingly, compared to E1162 wild-type, only *esp*, in addition to *ebrB*, was significantly down-regulated (2.3 fold) in E1162Δ*ebrB*. qRT-PCR confirmed a 2-fold lower expression of *esp* (*p*<0.001) in E1162Δ*ebrB* using the same cDNA. These results indicate that EbrB is not a global regulator in *E. faecium*.

### *Esp* is part of an operon that is controlled by *ebrB*

**Figure 4:**
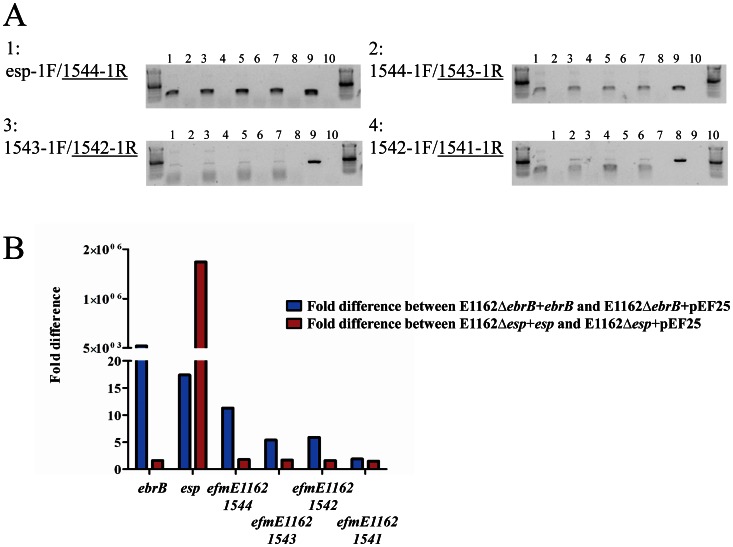
Determination of the *esp* operon. (A) RT-PCR analysis on synthesized cDNA on RNA obtained from wild-type E1162 using gene specific primers (underlined). Panel 1: intergenic region *esp* and *efmE1162_1544.* Panel 2: intergenic region *efmE1162_1544* and *efmE1162_1543.* Panel 3: intergenic region *efmE1162_1543* and *efmE1162_1542.* Panel 4: intergenic region *efmE1162_1542* and *efmE1162_1541.* RT-PCRs were performed on 4 biological replicates represented by lanes 1, 3, 5 and 7. Lanes 2, 4, 6, 8 represent negative controls in which incubation with reverse transcriptase was omitted. Lane 9 is the positive genomic DNA control and lane 10 negative water control. (B) Expression levels of *ebrB* and the *esp* operon in *ebrB* and *esp* complemented strains. Expression of *ebrB, esp, efmE1162_1544, efmE1162_1543, efmE1162_1542* and *efmE1162_1541* was analyzed using qRT-PCR on synthesized cDNA from the *ebrB* complemented strains (E1162▵*ebrB*+*ebrB* and E1162▵*ebrB*+pEF25) in blue and *esp* complemented strains (E1162▵*esp*+*esp* and E1162▵*esp+*pEF25) in red.

Analysis of the genetic organization of the *esp* region suggests that *esp* is part of an operon of in total five genes including *efmE1162_1544*, previously annotated as *nox* encoding a putative NADH oxidase, *efmE1162_1543*, previously annotated as *mur* encoding a putative muramidase, *efmE1162_1542*, previously annotated as *phage* encoding a hypothetical protein and *efmE1162_1541*, previously annotated as *permease* encoding a putative drug resistance transporter (Fig. 1B) [9]. To determine whether these genes are transcribed as a single RNA molecule, we performed intergenic PCRs on synthesized cDNA using gene specific primers on RNA obtained from E1162. In all cases PCR on genomic DNA was positive and as expected positive PCR results were obtained for the intergenic region between *esp* and e*fmE1162_1544* and e*fmE1162_1544* and e*fmE1162_1543*, although the latter signal was a little bit lower (Fig. 4A). A very weak PCR product was obtained for the intergenic region of genes e*fmE1162_1543* and e*fmE1162_1542*, which makes is dubious that e*fmE1162_1542* is indeed part of this operon. The fact that the intergenic region between e*fmE1162_1543* and e*fmE1162_1542* is larger than that between the more upstream located genes (349 bp compared to 134 bp and 112 bp, respectively, Fig.1B) and that RNAfold predicted a relative weak transcriptional terminator with a ▵G of -79.50 kcal/mol in the e*fmE1162_1543* and e*fmE1162_1542* intergenic region (Fig. 1B) also points towards the fact that e*fmE1162_1542* may not part of the *esp* operon. On the other hand, the weak PCR product we observed for the e*fmE1162_1543* and e*fmE1162_1542* intergenic region of genes could be the result of the fact that the RNA polymerase may read through this weak terminator or because of decline in expression due to degradation of a very large polycistronic mRNA (almost 10 Kb) that is transcribed from this operon, which may suggests that e*fmE1162_1542* is part of the *esp* operon. A positive though weak PCR product was also obtained between e*fmE1162_1542* and e*fmE1162_1541* (Fig. 4A), but this expression might be independent of EbrB as it is not likely that also this gene would be part of the operon based on the relative large intergenic region of 587 bp (Fig. 1B). In order to determine whether EbrB regulates this entire operon, we compared expression levels of *ebrB*, *esp*, e*fmE1162_1544*, e*fmE1162_1543*, e*fmE1162_1542* and e*fmE1162_1541* in the *ebrB* mutant strain complemented with wild-type *ebrB* (E1162Δ*ebrB*+*ebrB*), in which Esp expression was highly restored, with the *ebrB* mutant strain complemented with empty vector (E1162Δ*ebrB*+pEF25) using qRT-PCR on synthesized cDNA. As a control, the same qRT-PCRs were performed on synthesized cDNA isolated from the *esp* complemented strains E1162Δ*esp*+*esp* and E1162Δ*esp*+pEF25. As expected, *ebrB* and *esp*, encoded from multicopy plasmids in the complemented strains E1162Δ*ebrB*+*ebrB* and E1162Δ*esp*+*esp* were highly expressed compared to the strains complemented with empty vector pEF25, i.e. 4.7×10^4^-fold and 1.7×10^6^-fold, respectively (Fig. 4B). Interestingly, in the *ebrB* complemented strain overexpression of EbrB resulted not only in much higher expression levels of *esp* (16-fold), but also of the downstream encoded genes, i.e. e*fmE1162_1544* (11-fold)and e*fmE1162_1544*3 (5-fold) (Fig. 4B). As we also observed a 5-fold increased expression of e*fmE1162_1542*, it is not unlikely that also this gene is part of the *esp* operon. In contrast, only a<2-fold difference in expression was observed for ORF e*fmE1162_1541*, which is comparable with the fold difference identified in the *esp* complemented strain (Fig. 4B). If true, this decline in expression could be the result of degradation of the large polycistronic mRNA that is transcribed from this operon. In the *esp* complemented strains, there was, except for *esp,* no differential expression observed for *ebrB* and the downstream of *esp* encoded genes (all<2-fold). All together these data indicate that EbrB regulates not only *esp* but at least two and possibly three downstream located genes. The fact that we did not find differential expression of these three genes in our microarray experiments might be due to relative low transcription levels of *ebrB* in broth as also observed in our experiments where we assessed growth phase dependent expression of *ebrB* (Fig. 3C).

### Dynamics of biofilm formation

**Figure 5:**
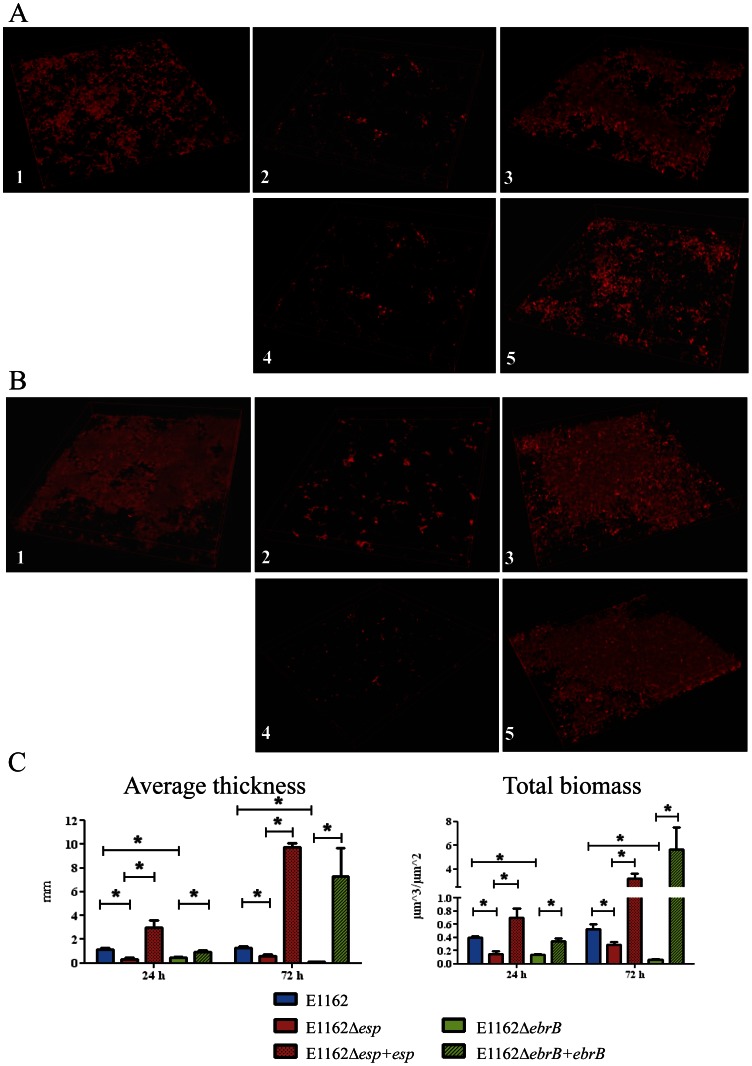
Biofilm semi-static model using confocal laser scanning microscopy (CLSM). Biofilms were grown in TSBg on coverslips coated with poly-L-lysin for (A) 24h and (B) 72h, including E1162 wild-type strain (1), *esp* mutant E1162Δ*esp* (2), the *esp* complemented strain E1162Δ*esp*+*esp* (3), *ebrB* mutant E1162Δ*ebrB* (4) and *ebrB* complemented strain E1162Δ*ebrB*+*ebrb* (5). (C) Quantification of the average thickness and total biomass of biofilms using Comstat/Matlab analysis. Asterisks represent significant differences (*p<0.01) as determined by an unpaired two-tailed Student's *t*-test) between the indicated samples. Pictures were taken at 63×magnification with 2.5 optical zoom.

Previously, Heikens *et al.* showed that Esp is involved in initial adherence and biofilm formation to polystyrene using E1162Δ*esp:cat*. Here we confirm abolished biofilm formation in the newly constructed *esp* markerless mutant, but also in the *ebrB* mutant using CLSM in a 24 h and 72 h semi-static biofilm model (Fig. 5A and B). Comstat/Matlab analysis revealed significant decreased average thickness (*p*<0.01) and total biomass (*p<*0.01) for both mutants at 24 h and 72 h, although at 72 h the decrease in biofilm formation in the *ebrB* mutant seemed to be more pronounced than in the *esp* mutant (Fig. 5C). Biofilm formation was restored even to higher levels than wild-type in the *esp* and *ebrB* complemented strains (Fig.5), while biofilm formation of the controls (*esp* and *ebrB* mutant complemented with the empty vector) was comparable to the respective *esp* and *ebrB* mutant strains (data not shown). Furthermore, a difference in biofilm composition can be observed between wild-type and complemented strains (Fig. 5A and B). In the biofilms of both complemented strains spots with higher intensities can be observed suggesting cell aggregation. Interestingly, a similar phenomenon was also observed when growing the strains in TSBg broth. After overnight growth both complemented strains appear to aggregate, while this was not observed in mutants complemented with the empty vector or with all strain grown in BHI broth (Fig. S2). This phenomenon is likely due to overexpression of Esp in the complemented strains. Because of the clear biofilm deficient phenotype of E1162▵*ebrB* we refer to this AraC-type regulator here as EbrB, for **E**nterococcal **b**iofilm **r**egulator **B**.

**Figure 6:**
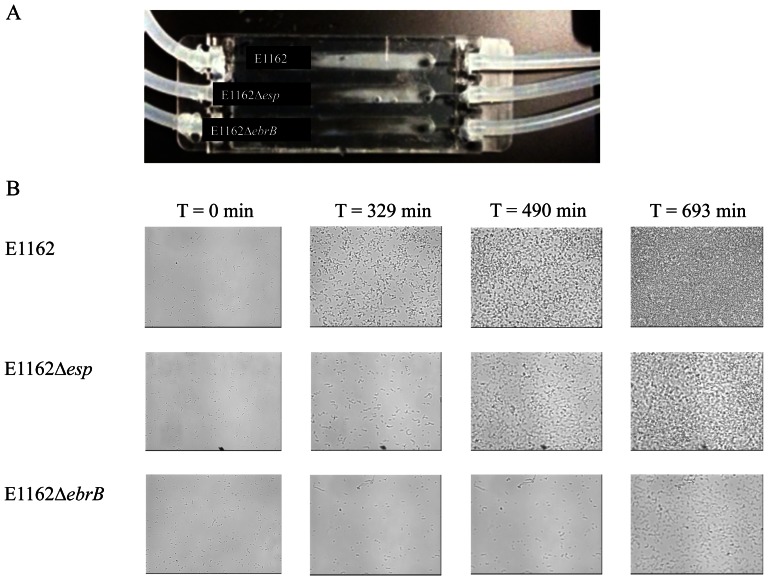
Biofilm formation using a flow cell system. Wild-type strain (E1162), *esp* mutant (E1162Δ*esp*) and *ebrB* mutant (E1162Δ*ebrB*) were grown in TSB diluted in PBS (1:10, v:v) with 1% glucose in a flow cell system. (A) Flow chambers after 17 h of biofilm formation using Stovall flow cell system. (B) Pictures at predefined position of time point zero and time points when start of biofilm formation for individual strains was observed, i.e. for E1162 at 329 min, for E1162▵*esp* at 490 min and for E1162▵*ebrB* at 693 min.

Dynamics of biofilm formation of strains wild-type E1162, E1162Δ*esp* and E1162▵*ebrB* were further investigated using flow cells. Although some biofilm formation was observed for all strains, visual inspection of the flow chambers after 17 h of growth clearly revealed the highest amount of biofilm formation for wild-type strain E1162 (Fig. 6A). Furthermore, for wild-type strain E1162 development of biofilm formation was observed after ∼329 min, while biofilm formation of the *esp* and *ebrB* mutants was less and clearly delayed at ∼490 min and ∼693 min, respectively (Fig. 6B). Also here, biofilm formation in the *ebrB* mutant seemed to be more attenuated than in the *esp* mutant. This may be due to the fact that EbrB also affects expression of the four genes located downstream of *esp*. The observed difference is likely not due to differences in growth rates as the growth curves were highly comparable when grown in TSBg (Fig. S1).

### Intestinal colonization

**Figure 7:**
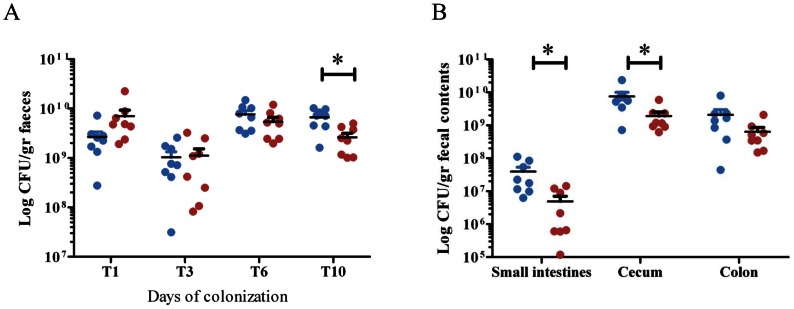
Intestinal colonization. Mice were orally inoculated with E1162 (blue dots) and *ebrB* mutant (E1162Δ*ebrB,* red dots). (A) Numbers of E1162 and E1162Δ*ebrB* were determined in stool of mice at different time point after *E. faecium* inoculation. (B) After 10 days of colonization numbers of E1162 and E1162Δ*ebrB* were determined in the small intestines, cecum and colon. Data are expressed as CFU per gram of stool/fecal contents and means are shown for 8 mice per group. Asterisks represent significant differences (*p<0.01) as determined by an unpaired two-tailed Student's *t*-test) between the indicated samples.

Next we investigated whether the *ebrB* mutant was attenuated in a mouse model of intestinal colonization. Both the wild type and the *ebrB* mutant were able to colonize the intestinal tract in comparable high numbers until day 6 after inoculation (Fig. 7A). Ten days after inoculation, a significant difference was observed between E1162 and E1162Δ*ebrB* with slightly but significantly higher CFU counts for the wild-type strain (7.2 (4.4 – 10.0)×10^9^ CFU/gram feces) than for the *ebrB* mutant (2.4 (1.0 – 5.0)×10^9^ CFU/gram feces) (*p* = 0.004) (Fig. 7A). Furthermore, significant lower amounts of E1162Δ*ebrB* compared to E1162 were present in the small intestine (1.4 (0.1 – 14)×10^6^ CFU/gram and 2.0 (0.6 – 11)×10^7^ CFU/gram, *p* = 0.02, respectively) and cecum (1.7 (0.6 – 5.9)×10^9^ CFU/gram and 6.1 (0.7 – 24)×10^9^ CFU/gram, *p* = 0.04) (Fig. 7B). Lower amounts of E1162Δ*ebrB* were also observed in de colon (4.3 (1.5 – 21)×10^8^ CFU/gram) compared to wild-type (15 (0.4 – 80)×10^8^ CFU/gram) although this difference is not significant (*p* = 0.13) (Fig. 7B). These results suggest that EbrB is also implicated in intestinal colonization.

## Discussion

Esp is a surface protein of *E. faecium* that is contained on the integrative conjugative element ICE*Efm1*, which is specifically found in hospital-associated *E. faecium* lineages. Previously, it has been demonstrated that Esp was involved in biofilm formation and virulence [12]–[14], [30]. In the current study, we identified a regulator of *esp*, designated EbrB for enterococcal biofilm regulator B, which is located just upstream of *esp* and characterized its role in Esp expression, biofilm formation, and intestinal colonization. Furthermore, we showed that *esp* is part of an operon that includes at least two and possibly three additional downstream located genes.

Esp of both *E. faecium* and *E. faecalis* belongs to the Alp family of proteins, where Alp stands for α-like proteins, which are characterized by the presence of long, completely identical repeats [35]. Alp proteins were first identified in *Streptococcus agalactiae*, including the α, Rib, R28 and Alp2 proteins, but were later also identified in other gram positive bacteria like R28 in *Streptococcus pyogenes* and Bap in *Staphylococcus aureus* (see Lindahl *et al.* [36] for a review on these proteins). So far nothing is known about transcriptional regulation of the genes encoding this group of proteins. Remarkably, the availability of whole genome sequences revealed that several of these Alp proteins (e.g. Rib and Alp2 in *S. agalactiae*) are located either up- or downstream of an AraC-type of regulator. In 47/53 *esp* containing *E. faecium* whole genome sequences (date December 2012), an AraC-type of regulator is located upstream of *esp*. In the remaining 6 *esp* containing whole genome sequences, this regulator was also present, but on the border of a contig. It is very likely that also in these strains this regulator is located upstream of *esp.* Therefore, it has been suggested that these proteins have a role in the transcriptional regulation of expression of these genes [36]. We, now, demonstrate for the first time that EbrB, a protein that contains the characteristic C-terminal HTH of an AraC-type of regulator, regulates expression of the Alp family protein Esp in *E. faecium*.

A BLAST search revealed that EbrB had the highest identity (60% AA) with the *E. faecalis* pathogenicity island encoded regulator, PerA, which is also located adjacent to *esp*, though downstream located [37]. Recently the regulon of the *E. faecalis* PerA was studied by comparing the transcriptome of an *E. faecalis* wild-type strain (E99) with its isogenic *perA* insertion mutant (DBS01) using microarray and qRT-PCR [37]. Despite the fact that PerA and EbrB display 60% similarity and are both *araC*-type regulator genes located adjacent to *esp* our transcriptome results for *ebrB* are different to those found for *perA*. Microarray analysis revealed that EbrB regulates only one gene, *esp*, while additional qRT-PCR experiments revealed that EbrB also controls expression of three additional genes located just downstream of *esp* on the *esp* containing ICE*Efm1* of *E. faecium* and that are part of a single operon. In contrast, microarray analysis of PerA identified in total 151 differentially expressed genes in mid- and late exponential and stationary phase, which are mainly encoded outside the *esp* containing pathogenicity island of *E. faecalis*. Consequently, PerA was suggested to act as global regulator and importantly PerA did not seem to control *esp* expression [37]. We cannot exclude that also other genes might be regulated by EbrB, but were not detected using microarray analysis due to low level of *ebrB* expression at OD_660_ 0.3.

To investigate involvement of EbrB in regulation of *esp* expression, we constructed an EbrB deficient mutant and found that *esp* expression, based on microarray and qRT-PCR, as well as Esp protein expression and surface exposure of Esp was reduced in the mutant and that this phenotype could be restored by complementing the mutant with full length EbrB. The reduction in Esp surface exposure in the EbrB deficient mutant was comparable with that observed in a newly constructed markerless E1162▵*esp* mutant. Also in the natural *ebrB* mutant *E. faecium* strain E0305, complementation with *ebrB* resulted in increased Esp expression levels. These experiments demonstrate involvement of EbrB in *esp* expression, but not direct binding of EbrB to the promoter of *esp* or its own promoter. This is currently under investigation. Sequence analysis of the promoter region did not identify an obvious EbrB binding site.

Previously, temperature and air oxygen dependent cell surface Esp expression was studied using plate grown cells [15]. Here, we investigated growth phase dependent cell surface expression of Esp using different growth media and showed a clear growth-phase dependent Esp expression in TSBg broth and not in BHI broth, though expression levels were much lower than in a high cell density condition like plate grown cells. This suggests that Esp surface exposure is dependent on cell density, which may indicate involvement of quorum sensing systems. In contrast to *E. faecalis* where an *fsr* quorum sensing system has been studied in detail [38], quorum sensing systems have never been described in *E. faecium* [39]. Because of this and the fact that only small fluctuations were observed for *ebrB* expression during different growth-phases, it is still unclear how exactly cell density dependent expression of Esp expression is regulated. EbrB is, however, necessary for basal expression while Esp is completely absent in all growth phases and in plate grown cells in E1162▵*ebrB*. It would be of interest to further investigate the existence of quorum sensing systems in *E. faecium* as they, like in *E. faecalis*, often play a role in virulence [38]–[43].

In the semi-static biofilm model and the flow cell system, differences were observed in biofilm formation between wild-type, mutants and complemented strains. In the semi-static model differences were more pronounced after 72 h of growth. In the flow cell model biofilm formation was observed in the wild-type strain and to lesser extent also in mutant strains. Furthermore, biofilm development in the mutant strains was delayed compared to wild-type. These data demonstrate a role for EbrB in early and later biofilm. From the Comstat analysis, including total biomass and average thickness, it is clear that biofilm formation in E1162Δ*ebrB* is more affected than in E1162Δ*esp*. A similar effect was observed using the flow cells. In addition, in the complemented strains more biofilm formation was observed in the *ebrB* complemented strain compared to the *esp* complemented strain. An explanation for this more pronounced effect of EbrB on *E. faecium* biofilm formation, relative to Esp, may reside in the fact that EbrB also controls expression of additional three genes located just downstream of *esp* on the *esp* containing ICE*Efm1* of *E. faecium*. Two of these genes encoding a putative NADH oxidase and a putative muramidase and it has been shown previously that proteins belonging to these classes are implicated in biofilm formation [9], [44]–[47].

In a mouse intestinal colonization model the *ebrB* mutant was clearly attenuated. Given the fact that in the *ebrB* mutant *esp* expression is affected resulting in the absence of Esp surface exposure, this observation seems to contradict with previous findings of Heikens *et al.* that based on a comparison of colonization capacities of an insertion-deletion E1162Δ*esp:cat* mutant with wild-type E1162 concluded that Esp is not essential in intestinal colonization of mice [30]. In fact an unexplained higher number of E1162Δ*esp:cat* was found in the small intestine. An explanation for these contradicting results might be that in the current study a different decolonization regime was used, resulting in higher colonization rates. However, another plausible explanation is that additional genes located downstream of *esp* and also controlled by EbrB contribute to colonization. The exact role of the genes located downstream of *esp* and contained on ICE*Efm1* in biofilm formation and colonization remains to be investigated.

In conclusion, we identified EbrB as a regulator of *esp* in *E. faecium* and its downstream encoded genes. Based on our current and previous [15] observations that Esp expression is growth condition dependent, indicates that expression of *esp* is regulated by environmental signals likely through EbrB. Regulation of AraC-type regulators by environmental signals and the effect of this on virulence gene expression is well documented and recently nicely reviewed [48]. Furthermore, we determined that during growth Esp is increasingly expressed at RNA and protein level, with highest expression in stationary phase which suggests involvement of an unidentified quorum sensing like system in regulation of *esp* expression in *E. faecium*. Understanding how virulence gene expression is regulated in *E. faecium* may foster the development of compounds that block the action of virulence gene regulators, like *ebrB*, which would impact on gut intestinal colonization and biofilm formation of specific multidrug resistance *E. faecium* hospital lineages that are enriched in the *esp* containing ICE*Efm1*.

## Supporting Information

Figure S1 **The effect of targeted mutations of** ***esp*** **and** ***ebrB*** **on growth of** ***E. faecium*** **.** Overnight cultures of wild-type, mutants and complemented *E. faecium* were inoculated at an initial cell density of OD_660_ 0.0025 in BHI or TSBg. Growth curves of strain E1162, the different mutant strains (panel A: BHI Δ*esp*; panel B: BHI Δ*ebrB*; panel C: TSBg Δ*esp*; panel D: TSBg Δ*ebrB*) and *in trans* complemented strains are shown. Growth curves are mean data of three independent experiments.
(TIF)

Figure S2 **Aggregation of *esp* and *ebrB* complemented strains.** Pictures of overnight in TSBg (panel A) and BHI (panel B) grown E1162Δ*esp* complemented with the empty vector pEF25 and pEF25+*esp* and E1162Δ*ebrB* complemented with the empty vector pEF25 and pEF25+*ebrB.* In the *esp* complemented strain E1162▵*esp*+*esp* grown in TSBg and to a lesser extent in the *ebrB* complemented strain E1162▵*ebrB*+*ebrB* cells have aggregated and form a sediment on the bottom of the tube. Mutants complemented with the empty vector grown in TSBG and all strains grown in BHI produced a more turbid, planktonic growth pattern.
(TIF)
