# Supplementary figures and images for: The Enterococcus faecium Enterococcal Biofilm Regulator, EbrB, Regulates the esp Operon and Is Implicated in Biofilm Formation and Intestinal Colonization
Source: PLoS One. 2013 May 31;8(5):e65224. doi: 10.1371/journal.pone.0065224 (PMC3669262; doi:10.1371/journal.pone.0065224)

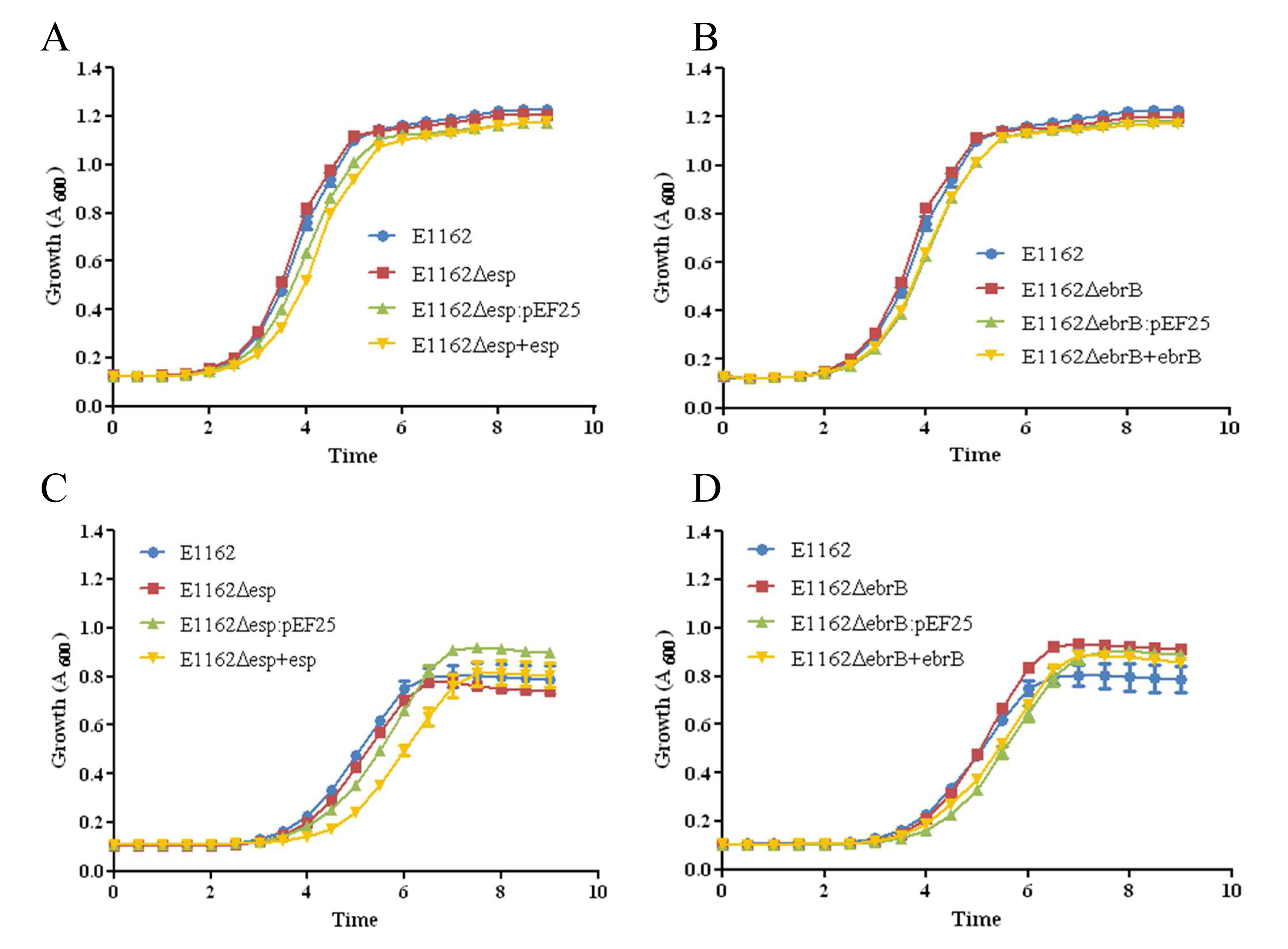

Supplement: Figure S1 — The effect of targeted mutations of esp and ebrB on growth of E. faecium . Overnight cultures of wild-type, mutants and complemented E. faecium were inoculated at an initial cell density of OD660 0.0025 in BHI or TSBg. Growth curves of strain E1162, the different mutant strains (panel A: BHI Δesp; panel B: BHI ΔebrB; panel C: TSBg Δesp; panel D: TSBg ΔebrB) and in trans complemented strains are shown. Growth curves are mean data of three independent experiments. (TIF) [file pone.0065224.s001.tif]

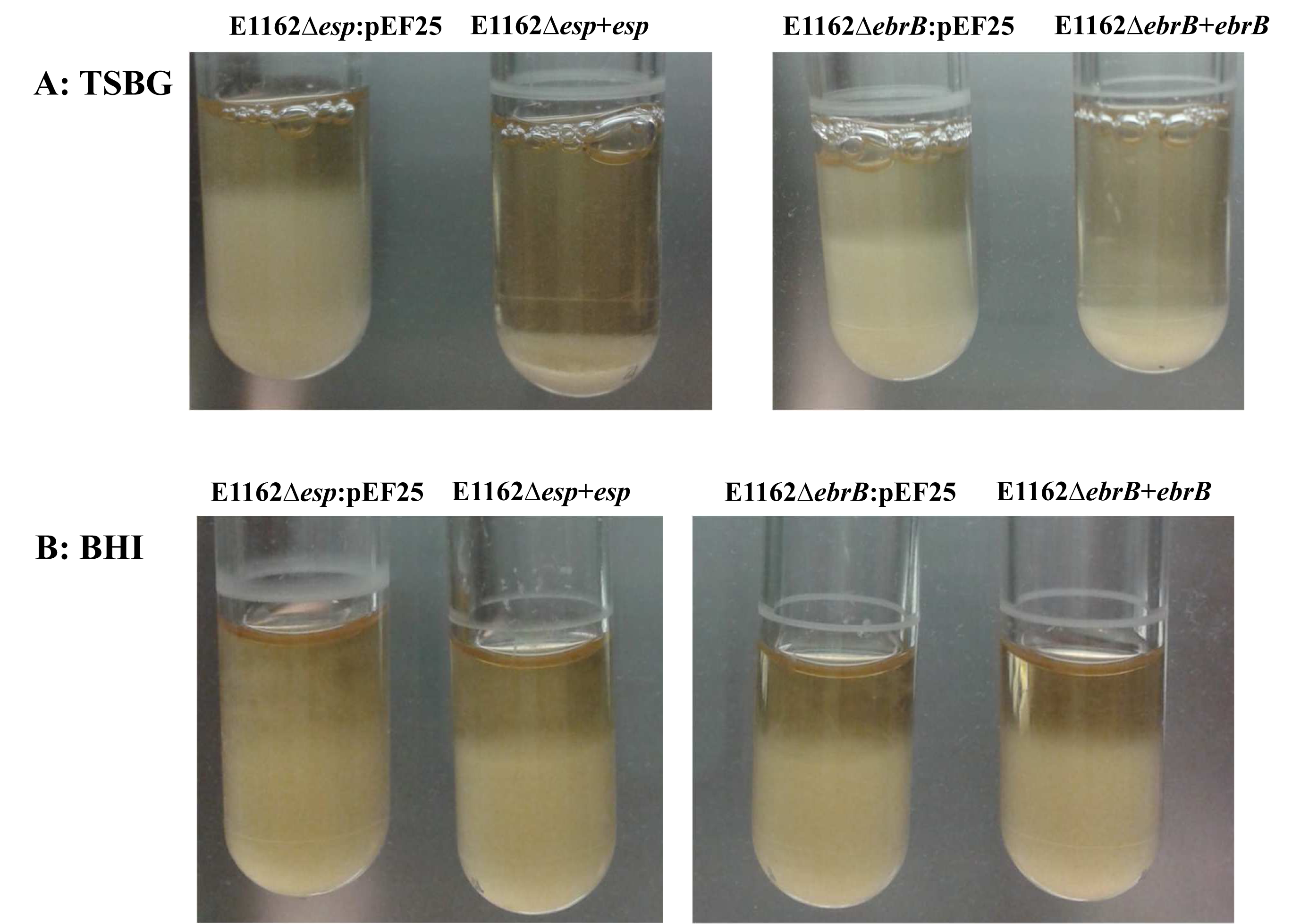

Supplement: Figure S2 — Aggregation of esp and ebrB complemented strains. Pictures of overnight in TSBg (panel A) and BHI (panel B) grown E1162Δesp complemented with the empty vector pEF25 and pEF25+esp and E1162ΔebrB complemented with the empty vector pEF25 and pEF25+ebrB. In the esp complemented strain E1162▵esp+esp grown in TSBg and to a lesser extent in the ebrB complemented strain E1162▵ebrB+ebrB cells have aggregated and form a sediment on the bottom of the tube. Mutants complemented with the empty vector grown in TSBG and all strains grown in BHI produced a more turbid, planktonic growth pattern. (TIF) [file pone.0065224.s002.tif]
